# Scales and Tooth Whorls of Ancient Fishes Challenge Distinction between External and Oral ‘Teeth’

**DOI:** 10.1371/journal.pone.0071890

**Published:** 2013-08-12

**Authors:** Qingming Qu, Sophie Sanchez, Henning Blom, Paul Tafforeau, Per Erik Ahlberg

**Affiliations:** 1 Subdepartment of Evolution and Development, Department of Organismal Biology, Uppsala University, Norbyvägen, Uppsala, Sweden; 2 European Synchrotron Radiation Facility, Grenoble, France; Monash University, Australia

## Abstract

The debate about the origin of the vertebrate dentition has been given fresh fuel by new fossil discoveries and developmental studies of extant animals. Odontodes (teeth or tooth-like structures) can be found in two distinct regions, the ‘internal’ oropharyngeal cavity and the ‘external’ skin. A recent hypothesis argues that regularly patterned odontodes is a specific oropharyngeal feature, whereas odontodes in the external skeleton lack this organization. However, this argument relies on the skeletal system of modern chondrichthyans (sharks and their relatives), which differ from other gnathostome (jawed vertebrate) groups in not having dermal bones associated with the odontodes. Their external skeleton is also composed of monoodontode 'placoid scales', whereas the scales of most early fossil gnathostomes are polyodontode, i.e. constructed from several odontodes on a shared bony base. Propagation phase contrast X-ray Synchrotron microtomography (PPC-SRµCT) is used to study the polyodontode scales of the early bony fish *Andreolepis hedei*. The odontodes constructing a single scale are reconstructed in 3D, and a linear and regular growth mechanism similar to that in a gnathostome dentition is confirmed, together with a second, gap-filling growth mechanism. Acanthodian tooth whorls are described, which show that ossification of the whorl base preceded and probably patterned the development of the dental lamina, in contrast to the condition in sharks where the dental lamina develops early and patterns the dentition.The new findings reveal, for the first time, how polyodontode scales grow in 3D in an extinct bony fish. They show that dentition-like odontode patterning occurs on scales and that the primary patterning unit of a tooth whorl may be the bony base rather than the odontodes it carries. These results contradict the hypothesis that oropharyngeal and external odontode skeletons are fundamentally separate and suggest that the importance of dermal bone interactions to odontode patterning has been underestimated.

## Introduction

**Figure 1:**
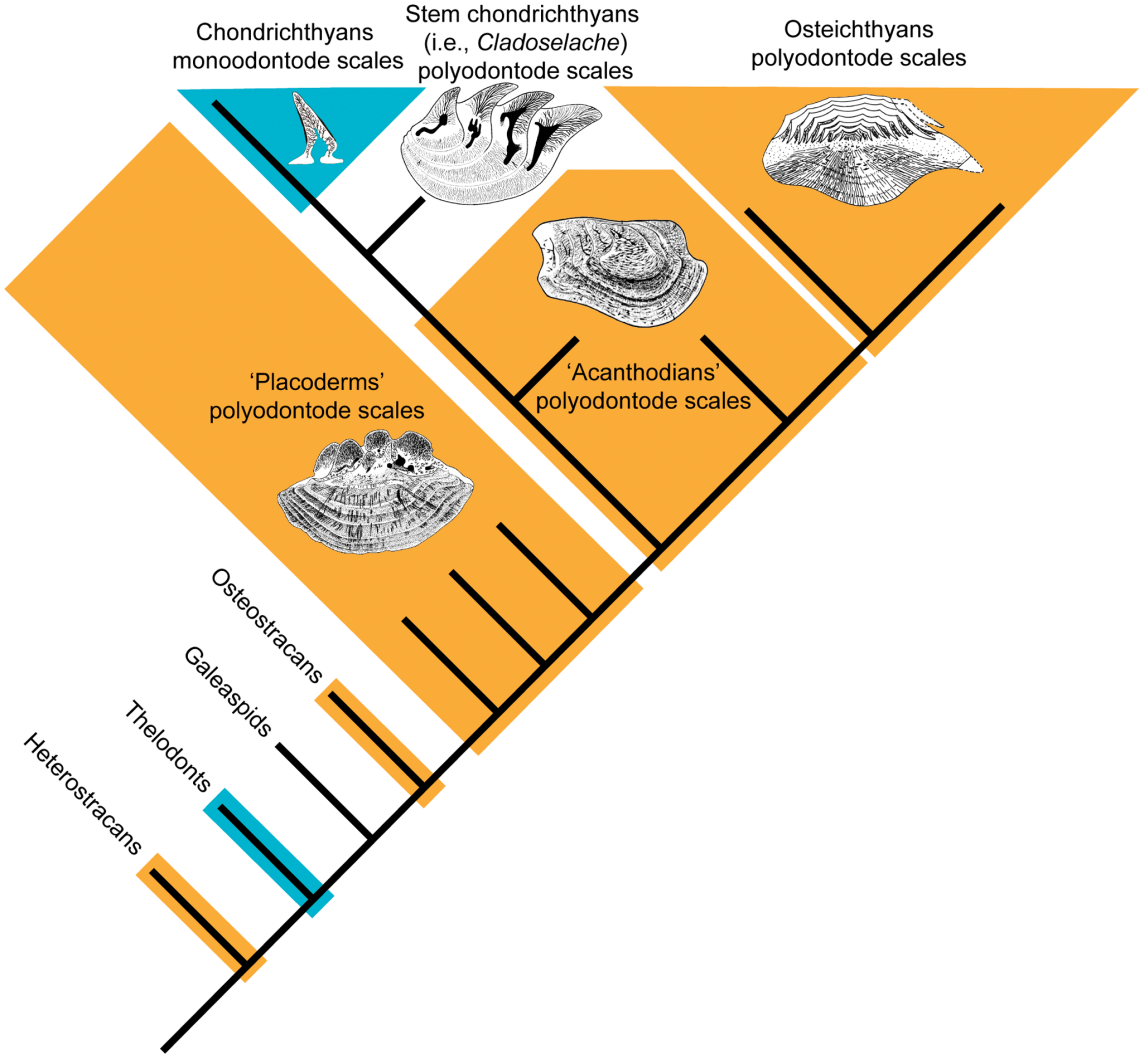
A simplified phylogeny of jawed vertebrates modified from Brazeau [14]. ‘Placoderms’ and ‘Acanthodians’ are extinct, probably paraphyletic, groups. Representative scales of osteichthyans (*Cheirolepis*), acanthodians (*Nostolepis*), stem chondrichthyans (*Cladoselache*) and ‘placoderms’ (*Ohiolepis*) are modified from [52], [53], [44] and [54] respectively.

A dermal skeleton composed of odontodes, discrete dentine structures (sometimes covered with enamel or enameloid) that develop around a mesenchymal papilla in contact with an overlying epithelium, is primitively present both on the external body surface and in the oro-pharynx of jawed vertebrates. However, in most extant representatives of the group the dermal odontode skeleton has been lost, leaving the teeth and/or pharyngeal denticles as the only remaining dentine elements in their anatomy. One major exception to this rule is the Chondrichthyes (sharks, rays and ratfishes), which frequently have well-developed dermal odontode skeletons composed of placoid scales (Figure 1), as well as teeth in the jaws. Because they are readily available for study, sharks have been used extensively to discuss problems related to the origin of the dentition in jawed vertebrates [1]–[6]. Consistent differences between the spatial organization of dermal odontodes and teeth have given rise to the hypothesis that the oro-pharyngeal odontode skeleton has a unique pattern, independent of the dermal odontodes [7]. This oro-pharyngeal pattern, consisting of odontodes arranged into a successional iterative order and induced by the covering dental lamina or odontogenic band, has been considered as a diagnostic character of true teeth [2]–[6], [8]–[11]. The presence of a similar odontode pattern in fossils has been inferred to indicate the existence of a dental lamina or odontogenic band during their development [3], [12]. However, it is still debated whether an ordering polarized growth is a valid criterion for the definition of teeth, as similarly organized odontode structures exist in the dermal skin skeleton of some placoderms [13].

The growth pattern of the skeletal system of thelodonts, a group of extinct jawless members of the jawed vertebrate stem group (Figure 1) that possess micromeric dentine squamation like modern sharks, has recently been studied by synchrotron X-ray microtomography [14]. The pharyngeal denticles of the thelodont *Loganellia scotica* are shown to be organized into fused linear arrays, strongly reminiscent of the tooth whorls observed in many chondrichthyans and some osteichthyans, but quite different from the dermal scales of the same species. We thus have evidence that the patterning distinction between dermal and oro-pharyngeal skeletons predates the origin of jaws and true teeth, although the phylogenetic position of thelodonts suggests that the oro-pharyngeal patterning system may have arisen independently several times during vertebrate evolution [14].

A striking feature of these analyses is that most have been carried out on vertebrates that lack large dermal bones and have dermal skeletons formed exclusively by non-growing scales: each scale is made of a single odontode (thus known as a monoodontode scale) and may eventually be shed. This type of odontode skeleton is in fact unique to Chondrichthyes and Thelodonti (Figure 1). The other major groups of jawed vertebrates, ‘Acanthodii’, ‘Placodermi’ (both probably paraphyletic [15]) and Osteichthyes, all primitively have polyodontode scales (Figure 1), meaning that each scale is formed from multiple odontodes attached to a bony base. Such a scale grows larger and thicker during the life span of the fish and is not shed. Whereas modern chondrichthyans all have monoodontode scales, several kinds of polyodontode scales have been reported in early chondrichthyans [16]–[21]. Polyodontode scales have also been discovered in osteostracans and heterostracans, two groups of extinct jawless vertebrates that, like the thelodonts, are members of the jawed vertebrate stem group (Figure 1) [22], [23]. Taken together this evidence strongly suggests that polyodontode scales are primitive for jawed vertebrates and that monoodontode scales have evolved independently in thelodonts and chondrichthyans. Similarly, chondrichthyan teeth are not associated with dermal jawbones, but such bones are present in placoderms, osteichthyans and some acanthodians and may thus be primitive for jawed vertebrates.

Here we examine the supposed distinctness of the external and oro-pharyngeal patterning systems with reference to fossils of two early jawed vertebrates from the Silurian Period: scales of the osteichthyan *Andreolepis hedei* (Gotland, Sweden; Hemse Beds, approximately 422 million years old) and tooth whorls of an unidentifed acanthodian (Saaremaa, Estonia; Ohessaare Beds, approximately 417 million years old). *Andreolepis* is most probably a stem osteichthyan [24], [25] whereas the acanthodian may be a stem osteichthyan, a stem chondrichthyan or possibly a stem gnathostome [15], [26] (Figure 1). It is arguable that such a loosely defined, multiply paraphyletic taxon as ‘Acanthodii’ had best be discarded despite its long history of use. However, we are not presenting taxonomic or phylogenetic arguments about the status of acanthodians (beyond identifying them as early jawed vertebrates that do not fall into the chondrichthyan or osteichthyan crown groups), and thus feel justified in retaining the name as an informal label.

The scales of *Andreolepis* are polyodontode and the acanthodian tooth whorls have bony bases onto which the teeth are attached; both conditions are likely to be primitive relative to the monoodontode scales and tooth whorls without bony bases seen in crown-group chondrichthyans. Using propagation phase contrast X-ray synchrotron microtomography (PPC-SRµCT), we describe the complete three-dimensional distribution and growth pattern of odontodes in a polyodontode scale. Our results challenge the hypothesis of a rigid distinction between external and oro-pharyngeal odontode patterning.

## Materials and Methods

### Ethical Statement

The *Andreolepis* specimen was borrowed from the Stockholm Museum of Natural History. The material from Estonia was collected as part of collaboration with our colleague Tiuu Märss, Institute of Geology, Tallinn University of Technology. Dr Märss had permission from the local authorities (Saaremaa Bureau of the Hiiu-Lääne-Saare Region of the Estonian Environmental Board) during the collection of the material. As a rule, the specimens will be returned to Estonia after the study.

#### The scales of *Andreolepis*

Abundant *Andreolepis* scales were extracted from Late Silurian limestone from Gotland, Sweden, by dissolving the rock in dilute acetic acid [27]. Four scales were studied in total: one was scanned, three were sectioned. The scanned scale is a flank scale from the anterior part of the trunk [28].

#### Thin sectioning

**Figure 2:**
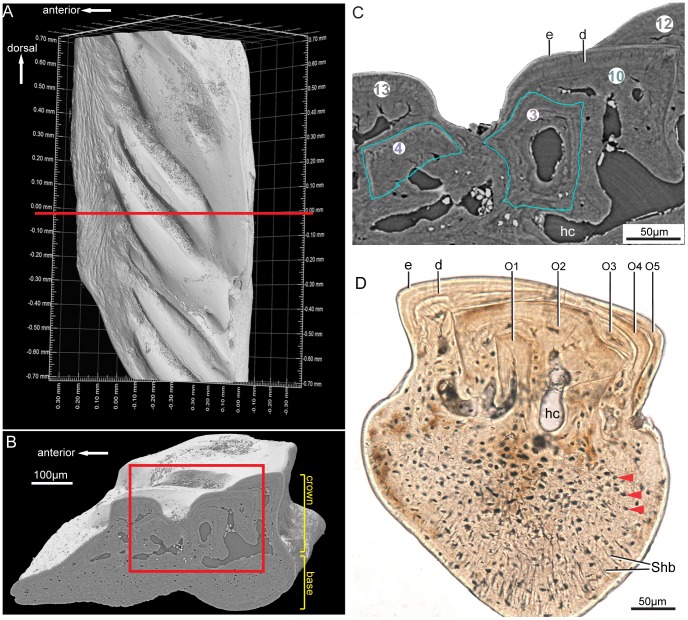
The scanned scale of *Andreolepis hedei* Gross, 1968. **A.** Scanned part of the scale (PMU 24786) rendered in VG Studio 2.1, crown view, red line marking the position of the slice in **B**. **B.** Longitudinal virtual thin section from the synchrotron scanning data, the red rectangle marking the region in **C**. **C.** Close-up of **B**, showing the segmentation of odontodes and the tissue composition of the crown (picture exported from VG Studio 2.1). The numbers in circles correspond to the odontodes in following Figures 3 and 4. **D**. A real thin section (PMU 24784) from an *Andreolepis* body scale made in the anteroposterior plane for comparison (DIC, optical microscopy); the scale is slender and comes from the posterior region of the body [28]. **Abbreviations: e**, enamel; **d**, dentine; **hc**, horizontal vascular canal, **O1-O5**, odontodes 1–5, **Shb,** Sharpey’s fibers.

Three scales were sectioned after embedding in resin. Sections were made in the longitudinal (i.e. anteroposterior) plane, in the middle region of the scale (Figure 2D). Thin sections were observed and photographed using transmitted and polarized light microscopy - Leica Photomicroscopy with Nomarski Differential Interference Contrast (DIC) - at the Department of Organismal Biology, Uppsala University.

#### Propagation phase contrast X-ray Synchrotron microtomography (PPC-SRµCT)

One scale was imaged at beamline ID19 of the European Synchrotron Radiation Facility (ESRF), France. The sample was scanned at 30 keV with a monochromatic beam, using a single crystal 2.5 nm period W/B4C multilayer monochromator. The beam was filtered with 2 mm of aluminium. The gap of the U32 undulator was closed at 12.38 mm. The detector used was a FreLoN 2K14 CCD camera coupled on a microscope optic that provides an isotropic voxel size of 0.678 µm. The scintillator used was a 10 µm-thick gadolinium gallium garnet (GGG) doped with europium [29]. In order to reveal the histological microstructures of the scale with phase contrast, the sample was fixed at a propagation distance of 30 mm from the detector. Two thousand projections were performed during continuous rotation over 180 degrees. The time of exposure per projection was of 0.3s. The isolated *Andreolepis* body scale scanned is about 2 mm long and 0.9 mm wide. As the field of view at high resolution is restricted to 1.4 mm, a small dorsal part and a small ventral part of the scale are missing (Figure 2A). The data obtained in edge detection mode were reconstructed using a classical filtered back-projection algorithm (PyHST software, ESRF). Segmentation and modeling were done using the software VG Studio 2.1 (Volume Graphics, Heidelberg).

#### Acanthodian tooth whorls

Numerous acanthodian tooth whorls, along with other vertebrate microremains, were extracted from Late Silurian limestone from Saaremaa, Estonia, by dissolving the rock in dilute acetic acid [30]. Two tooth whorls were selected for study: they represent an abundant morphology type (several tens of specimens in our sample) but other tooth whorl types that presumably represent other taxa are also present in the material. The two specimens were imaged using a Zeiss Supra 35-VP field emission SEM at the Department of Organismal Biology, Uppsala University.

## Results

### (a) *Andreolepis* Scales

**Figure 3:**
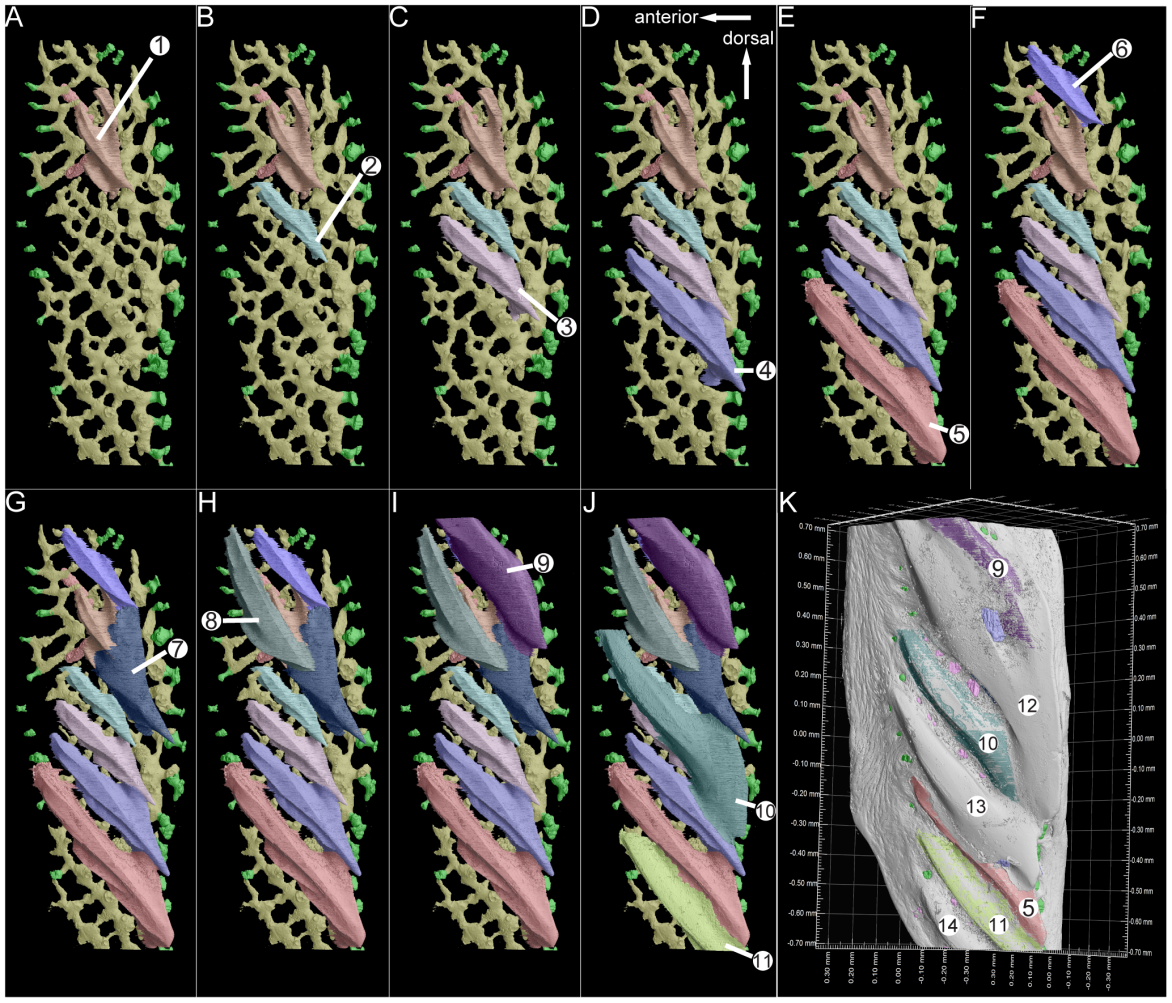
The reconstructed growth pattern of odontodes in the scanned scale of *Andreolepis*, crown view. **A–J.** The referred sequential addition of odontodes 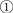 – 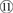 in the crown of the scale. The first generation odontodes (odontodes 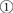 – 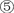, see text) form a growth series, but the other younger odontodes (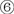 – 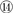) do not necessarily fall neatly into the same sequence even though they generally continue to get larger; the yellow horizontal vascular canal system is used as landmark to show the positions of the odontodes **K**. Crown view of the scale with buried odontodes, showing the actual surface composition of the scale. Note that the most dorsal denticles compose the enamel layers from both odontode 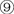 and 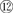, odontode 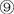 is partially overlapped by 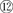; odontode 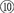 is only overlapped by 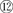 and 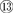 posteriorly and exposed to the surface otherwise.

**Figure 4:**
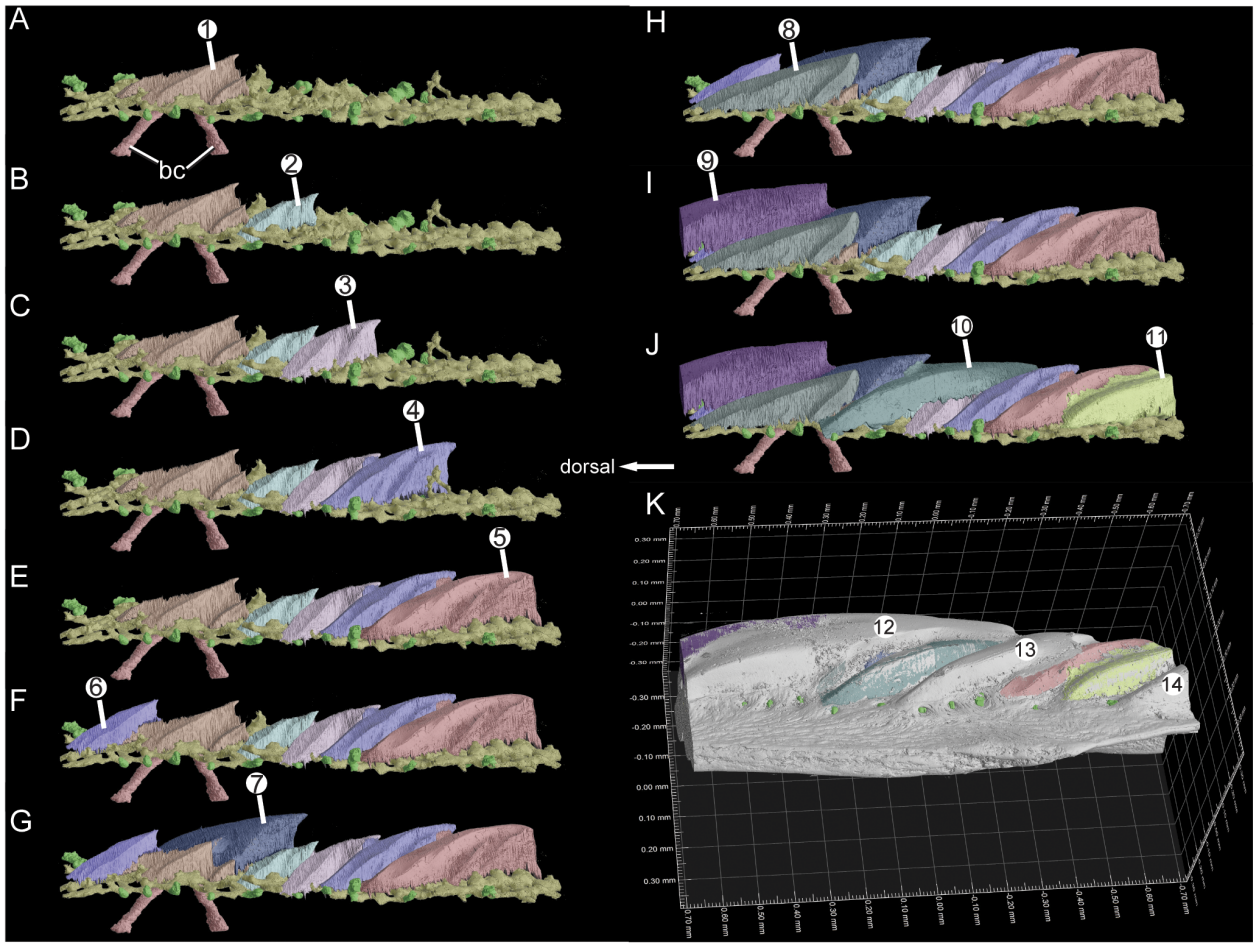
The reconstructed growth pattern of odontodes in the scanned scale of *Andreolepis*, anterolateral view. For explanations see Figure 3.

As already described by Gross [31] and Richter [32], the upper part (crown) of the *Andreolepis* scale consists of multiple odontodes (enamel and dentine fused to the basal bone) and the lower part (base) of cellular bone (Figure 2B–D; Figure S1). The high-resolution scan permits the observation of small structures such as osteocyte lacunae, Sharpey’s fibers and dentine tubules (Figure 2B, C; Figure S1) [33]. The boundary between two odontodes is always marked by the enamel layer of the buried odontode (Figure 2C); there is no sign of resorption of buried odontodes either in the scanned or sectioned specimens (Figure 2B–D; Figure S1; Movie S1). The surface of each odontode is thus represented by the surface of its enamel layer, allowing each odontode to be segmented and modeled in 3D (Figure 3, 4). As the horizontal vascular system marks the boundary between the dentinal and bony tissues, this system was also reconstructed as a landmark to show how odontodes are distributed above it (Figure 3). It is from the horizontal vascular system that pulp canals arise into each odontode. The bony base of the scale is pierced by two basal canals (in pink in Figure 4), which appear to represent the vascular loop around which the scale grew (see below).

14 odontodes can be identified in the tomographic data set, of which only 3 odontodes are fully exposed to the surface of the scale, while the other 11 odontodes (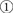
–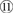 in Figures 3 and 4; Movie S2) are either partially or fully embedded inside the crown of the scale. Thus on the crown view of the scale, there are six denticles observable, but these consist of surface areas belonging to 7 odontodes (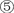 and 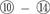). Although the most dorsal and largest denticle of the scale appears in external view to be a complete odontode with a rather flat surface (Figure 2A), it is in reality composed of two different odontodes 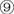 and 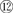 (Figures 3I, K); the sloping surface of odontode 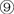 is partially covered by odontode 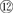 (Figure 3K). This is also the case for odontode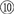, whose posterior tip is covered by odontode 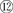 and 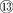 (Figures 3J, K). It is worth to mention that the scale was not fully scanned and two small parts have been missed. However, the missing parts are close to the dorsal and ventral ends of the scale respectively and will not affect our reconstruction results (see below).

**Figure 5:**
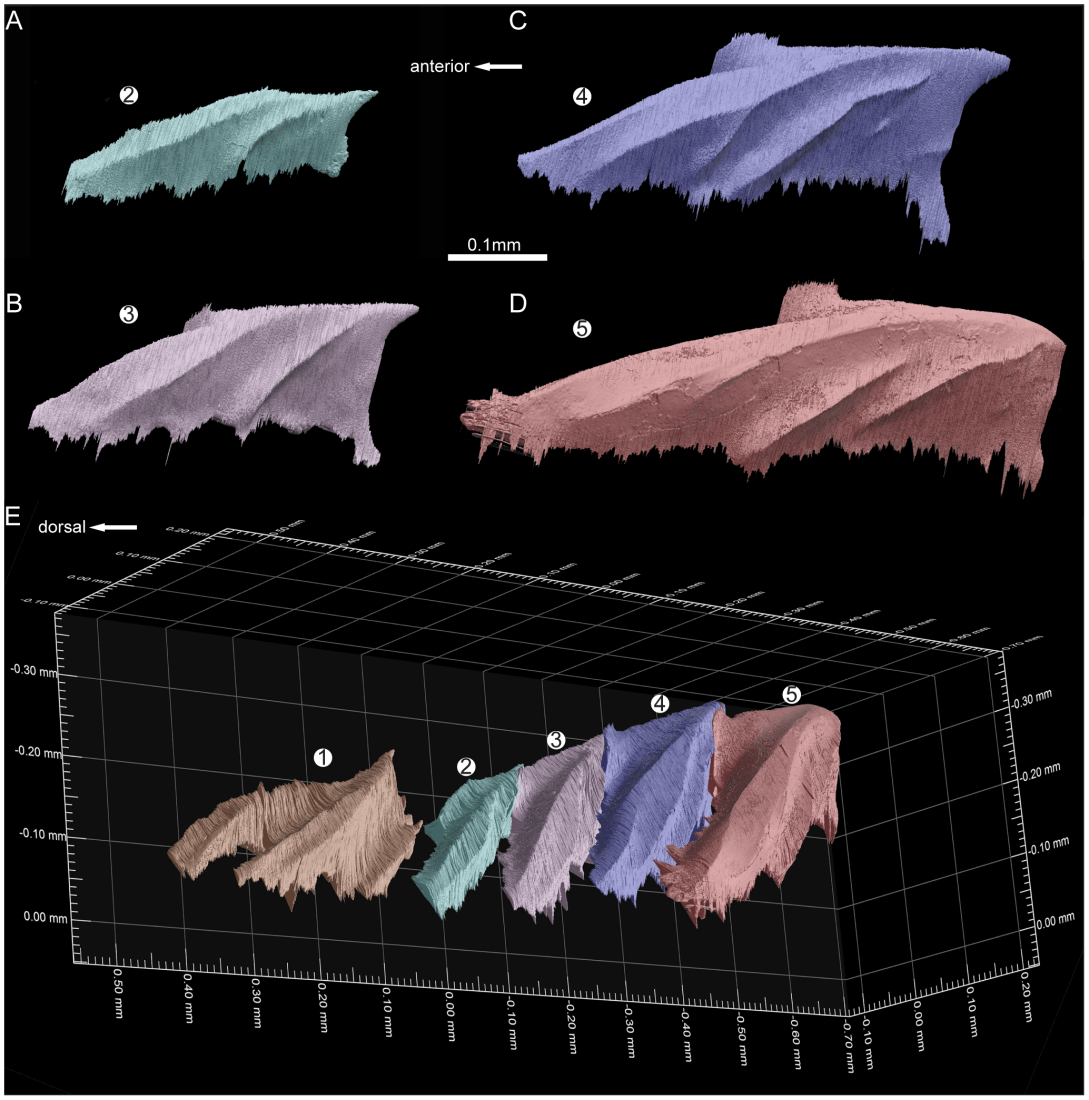
The reconstructed odontodes 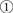
– 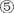 showing the consistent morphology and overlap relationships. **A–D.** Odontodes 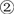 – 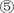 in ventral view. **E.** Odontodes 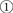 – 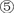 in anterodorsal view, in original relative positions showing the overlap mode of odontodes 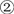 – 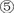.

Odontodes 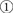 and 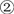 are the smallest, and two large basal canals (bc) pierce the bony base of the scale to connect with the horizontal vascular canal system just below odontode 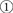 (Figure 4), indicating that odontode 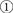 is the growth centre of the scale and presumably that it is the oldest odontode. Four odontodes (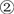 – 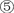 in Figure 3 and 4) distribute along the dorsoventral axis of the scale ventral to odontode 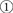, each one overlapping the ventral tip of its dorsal neighbor and being slightly larger (Figure 5E). These four odontodes exhibit similar surface morphology, although the larger odontodes have one or two more ridges on the ventral side (Figure 5A–D).

Surrounding odontode 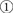 there are five odontodes (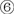 – 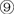 and 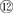 ) superimposing in the following way: the posteroventral part of odontode 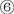 overlaps the dorsal part of odontode 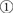; the anterodorsal part of odontode 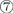 overlaps the posteroventral part of odontode 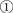 and the posteroventral edge of odontode 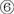; the posteroventral part of odontode 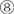 overlaps the anterodorsal part of odontode 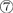 and the anterodorsal part of odontode 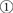; odontode 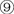 overlaps odontode 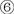 completely and the anterodorsal part of odontode 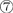; and the final odontode 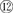 covers all these odontodes except the most superficial part of odontode 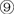. In this way, the scale crown grows both to be wider and thicker. Ventral to odontode 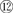, three odontodes (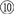, 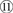, 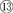) cover odontodes 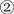 – 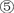 in a regular way, by filling the gaps between 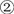 and 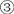 (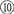), 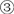 and 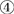 (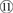), 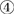 and 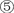 (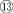). Odontode 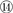 marks the most ventral part of the scan (Figure 3K).

### (b) Acanthodian Tooth Whorls

**Figure 6:**
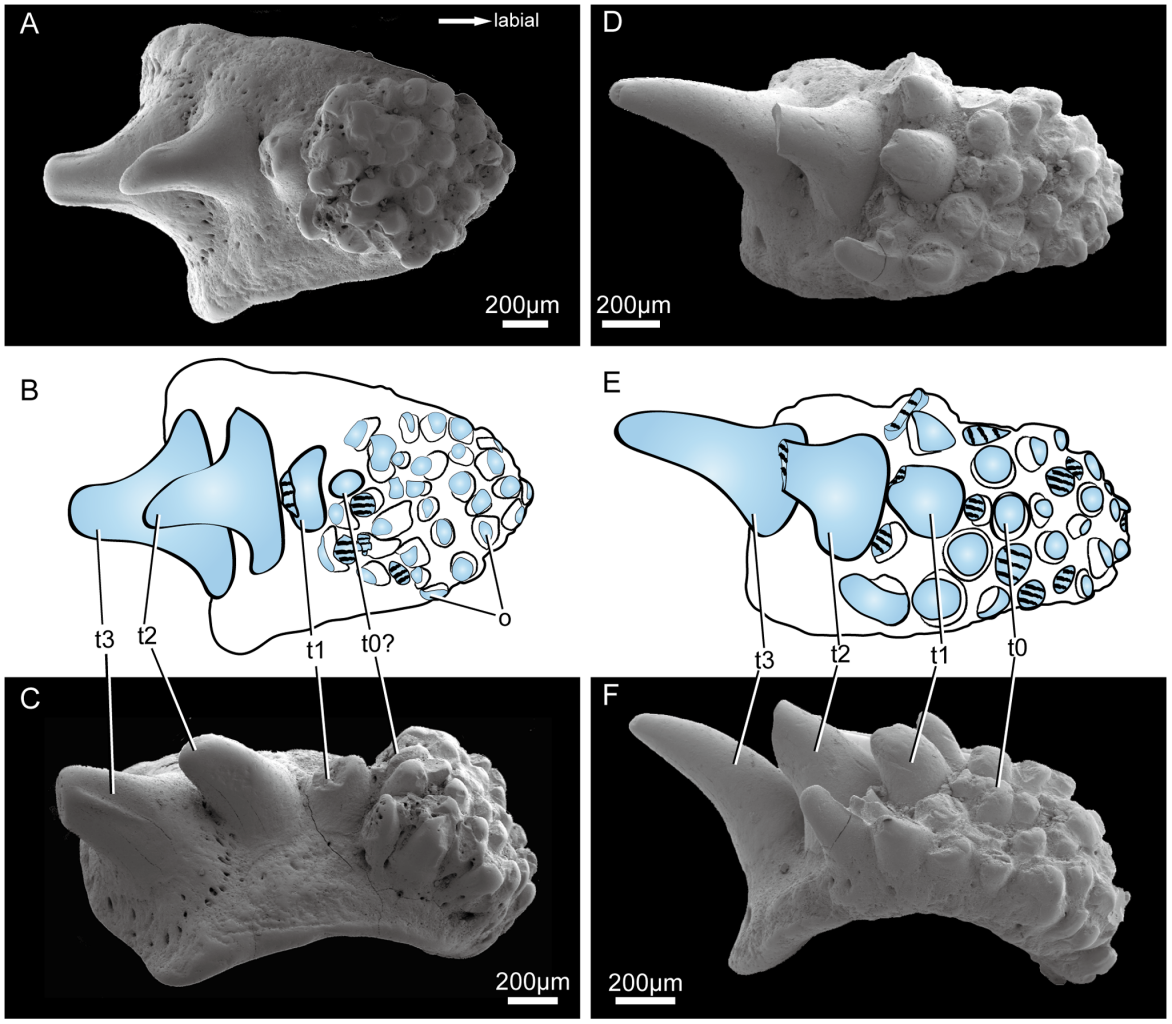
Two acanthodian tooth whorls (SEM photos with line drawings). **A–C,** GIT 658-1; **D–F,** GIT 658-2. **A,D.** Oral views, labial to the right. **B,E.** Sketch interpretations of A,D. **C,F.** Lateral views, labial to the right. **Abbreviations: t1-t3,** tooth element 1–3 belonging to the linear growth serial; **t0?,** possible origination element of the linear growth serial; **o,** disorganized odontodes on the labial base.

Each whorl consists of a bony basal plate with spiral curvature bearing a number of odontodes (Figure 6). The most labial (on the right in Figure 6 A–F), and therefore oldest, odontodes form a crowded and disorganized array of similar-sized blunt denticles that are not arranged into recognizable rows. This array occupies approximately 35% to 55% of the length of the oral surface of the whorl. The lingual (younger) part of the whorl carries three well-defined teeth (on the left in Figure 6 A–F), morphologically different from the denticles and all much larger, increasing in size from labial to lingual. They are arranged in a row running down the middle of the oral surface. The transition between these two regions differs between the two specimens. In GIT 658-1 (Figure 6A–C) it is abrupt, with the oldest tooth abutting against the lingual margin of the denticle field and no obvious size or shape gradient among the denticles. In GIT 658-2 (Figure 6D–F) it is gradual, with the oldest tooth surrounded by denticles and the most lingual denticles displaying increased size and a tooth-like morphology.

## Discussion

Based on thin sections, many studies have been carried out to describe the growth pattern of polyodontode scales in early jawed vertebrates [34]–[38]. Two major processes have been proposed to describe their growth pattern: areal growth (in an upward direction, causing the crown to become thicker) and superpositional growth (in dorsoventral or anteroposterior directions, causing the crown to become wider) [35], [39]. The thin section in Figure 2D indicates that odontode 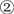 (O2) grows both superpositionally and areally relative to odontode 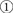 (O1). However, what these two growth modes mean in a 3D context cannot be addressed based only on 2D thin sections, and the true morphology of each odontode cannot be inferred from the surface of the scale. For example, the growth pattern of the scales of the early actinopterygian *Moythomasia* has been described as follows: “Initially there are separate ridges of dentine and ganoine … and new ganoine and dentine are added between the ridges until the whole external exposed surface is ganoine-covered” [40]. Although this conclusion is correct based on thin sections and surface structures of many scales, it does not explain the internal structure of the scales or how the different odontodes (‘ridges of dentine and ganoine’) are related to each other topologically. The reconstruction of the surface of each odontode in the *Andreolepis* scale clearly demonstrates that the morphology and growth pattern of the crown odontodes are much more complex than revealed by thin sections.

All the odontodes are oriented in an anterodorsal to posteroventral direction parallel to the ventral margin of the scale, with sharp points marking the posteroventral ends, giving each odontode the appearance of a posteroventrally sloped ‘tooth’ (Figures 3, 4 and 5). Odontodes 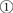 – 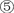, which probably represent the first generation of odontode deposition and grew directly on the bony base of the scale, exhibit a stereotypic morphology with one large ledge on the dorsal margin and several ridges (2–3) on the ventral face (Figure 5). The younger odontodes are morphologically more variable, reflecting the fact that they had to grow on top of preexisting odontodes that already occupied the space. Based on the above information, several observations could be made.

Firstly, only the first generation of odontodes (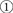 – 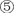) remains consistent in morphology with similar shape and ridge ornament, although the odontode 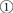 has larger ledge dorsally and ventrally As described above, the distribution of odontodes 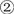 – 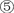 and their similar shape strongly suggest a growth process from dorsal to ventral, with each new larger odontode being added next to its dorsal neighbor and partly overlapping it in a repeated pattern. In this way the growth pattern of first generation odontodes can be described as areal growth, defined in terms of a three dimensional growth model. The most posteroventral part of the dorsal odontode is always overlapped by the dorsal ledge of the following ventral odontode (Figure 5E), and this overlapping pattern excludes the possibility that odontodes 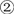 – 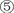 grew in a ventral to dorsal sequence. The distance between odontodes also remains consistent. Such a pattern implies the existence of a highly organized odontogenetic process, involving a persistent strip of odontode-producing tissue along the ventral edge of the scale that is activated in a stereotypic manner at regular time intervals. This type of organized odontogenetic program characterizes the dentition of gnathostome jaws, where the generative tissue would be described as an odontogenetic band or dental lamina [3], [7].

Secondly, subsequent generations of odontodes exhibit a gap-filling growth pattern. Because the first generation odontodes already occupy much of the surface area of the crown, younger odontodes have to grow into different shapes by overlapping those existing odontodes of different sizes. This is likely why younger odontodes (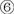 – 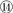) show much greater morphological variability than the first generation odontodes. The scale crown achieves its final thickness and areal extent through this gap-filling growth mechanism.

It should be mentioned that the sequence of addition of new odontodes cannot be inferred for those odontodes without overlap relationships. For example, the addition of odontode 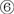 may have happened either before or after the growth series 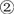 – 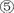. However, this does not affect the conclusion that an ordered growth pattern is present in odontodes 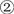 – 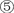.

Fraser and Smith [7] showed that, in modern sharks, teeth generated in an organized iterative sequence are only present in the oropharyngeal system, while the skin skeleton exhibits a random gap-filling growth pattern of independent odontodes or placoid scales. However, in the polyodontode scales of *Andreolepis* we find both the ordered pattern and the gap-filling growth pattern activated at different times and positions. Given that polyodontode scales appear to be primitive for jawed vertebrates (Figure 1), this suggests the possibility that ordered odontode generation could be a plesiomorphy in both the oropharynx and the skin skeleton of gnathostomes rather than a unique defining characteristic of ‘teeth’ [13].

The occurrence of organized tooth-like odontodes on scales, far away from the tissue boundaries and morphological architecture of the oropharynx, also raises the question of how these scale odontodes are patterned. The layout of the first generation odontodes in *Andreolepis* suggests a developmental relationship to the growing ventral edge of the scale, in other words a patterning link with the underlying dermal bone. The bony base of the *Andreolepis* scale is of a rhomboid type that is widespread among early osteichthyans and appears to be primitive for the osteichthyan crown group [22]. Such scales have a distinctive and quite stable morphology featuring a convex dorsal margin (sometimes developed into a peg), a convex ventral margin (sometimes developed into a socket) and a vertical ridge on the internal face [22], [28]. Primitively they bear an external covering of odontodes, as we see in *Andreolepis*, but in a few taxa such as *Panderichthys* the odontodes have been lost [41]. This loss does not produce any changes in the morphology of the bony basal plate, showing that the latter was patterned in its own right and not merely a passive by-product of odontode patterning. The developmental relationship between the odontodes and basal plate in *Andreolepis* was thus probably not one of odontodes simply inducing dermal bone formation. A feedback system involving regulatory signals passing in both directions between the odontode buds and the growing edge of the bony scale seems much more likely.

A similar patterning link between odontodes and bony base is also indirectly demonstrated by the acanthodian tooth whorls from the Ohessaare Beds in this study.

A typical acanthodian tooth whorl is composed of a spirally curved bony base with odontodes organized in a labio-lingual row, occasionally with smaller odontodes organized in side rows surrounding the main row [34], [42], [43], [44]. Except for the bony base the acanthodian tooth whorls closely resemble the tooth whorls and tooth families of chondrichthyans [15], [34] and there can be little doubt that they were generated in a similar manner by a dental lamina. In living chondrichthyans, tooth formation begins with the establishment of embryonic tooth germs at intervals along the jaw, and these in turn initiate the formation of tooth families [2]. A typical acanthodian tooth whorl could be explained in a similar way, with the added step that the presence of the teeth induces the formation of a bony basal plate. However, the tooth whorl morphology described here cannot be explained by such a scenario. The spiral curvature of the bony base shows that it was produced in the normal manner by addition of bone along the lingual margin. But during the first one-third to one-half of this growth process there was no associated progenitor tooth or organized tooth family: instead the oral surface of the whorl was simply covered with disorganized denticles. Only at the end of this early phase did an organized labio-lingual tooth row appear. This implies that the odontogenetic epithelium of the jaw margin initially was not developed into a dental lamina, and expressed no organized positional information. Only later did a dental lamina develop and begin to generate true teeth in specified positions. This implies in turn that the position of the tooth-generating sites was determined not by the spacing of progenitor tooth germs as in a modern chondrichthyan, but in relation to the preexisting bony bases of the tooth whorls. The actual patterning signal of the organized odontodes may have been generated by the dermal bone, or bone and tooth may both have responded to a signal from some other source, but it is clear that the bony bases were already patterned before teeth started to form in the appropriate locations.

Although typical acanthodian tooth whorls carry organized teeth throughout their growth history, this does not necessarily imply that development of their basal plates was simply induced by the teeth; initiation of tooth whorl formation by a signal from the bony base, or initiation of both bone growth and tooth whorl formation by a signal from another adjacent source, are alternative possibilities for these tooth whorls as well. Furthermore, a gradual transition from facial scales to teeth is present in some articulated ischnacanthid acanthodians [45], suggesting that a dental lamina emerged late in ontogeny in these forms. The patterning relationships indicated by the partly denticulated tooth whorls from Ohessaare may thus be representative for a wide range of acanthodians. Our results show that tooth-like organized sequential odontode development occurred on the scales of at least one stem osteichthyan, probably patterned in direct relationship to the bony base of the scale, and that the bony base rather than the tooth family or the dental lamina was likely the primary patterning unit in the tooth whorls of at least one acanthodian. These results both contradict the sharp distinction between oropharyngeal and skin odontode skeletons hypothesized on the basis of data from recent chondrichthyans [7], and further suggest a previously unrecognized role for the dermal bones in patterning the odontode skeleton. Although a distinct evolutionary origin of oropharyngeal and skin denticles has been refuted in previous work [46]–[48], no previous study has explicitly shown a tooth-like pattern of odontode addition in the external skeleton. Young [13] suggested that this tooth-like pattern of odontode addition may exist in dermal skin skeleton (e.g., fin spines) of some placoderms based on surface morphology, but the histology of the relevant material is still unknown. The supposed lack of such a pattern in the external skeleton has been a key point used by Fraser and Smith [7] to maintain their hypothesis that the oropharyngeal and dermal odontode systems are fundamentally distinct. The question is to what extent our results from *Andreolepis* are generally applicable to early jawed vertebrates. Most such vertebrates possess polyodontode scales, but because their histology has been studied only by traditional 2D thin sections we know little about their growth modes. For example, there are also deeply buried odontodes in several placoderms according to their thin sections [49], but comparison with the growth patterns presented here cannot be carried out until 3D reconstructions have been produced from high resolution scan data allowing 3D virtual paleohistology. The polyodontode scales of the primitive chondrichthyan *Cladoselache* cf. *fyleri* were described as showing a regular areal growth pattern [21], [44], while the growth mode of many [34] but not all [15], [50] ‘acanthodian’ scales has been described as ‘onion-like’. Although thin sections are rather reliable sources to study the tissue composition of the dermal skeleton, only 3D reconstruction can tell us the growth pattern in relative time and space. All these polyodontode scales of early jawed vertebrates, together with those of jawless vertebrates, will need to be reexamined in 3D on the basis of high-resolution scan data in order to properly address questions relating to their growth, development and patterning.

The hypothesis of fundamentally distinct oropharyngeal and external odontode skeletons has been linked to a mechanistic argument that the two are dependent respectively on endo- and ectodermal signaling [2], [5], [7], [9], [11]. However, recent cell lineage labelling experiments have shown that the teeth of mouse receive no endodermal contribution [51], suggesting that the cell population identity of the epithelium is not a determinant factor for the type of odontode patterning. This result is consonant with our fossil data, and leads us to predict that a relatively simple set of shared odontode patterning mechanisms and growth modes will prove to be responsible for the wide range of odontode patterns observed both in the external and in the oropharyngeal skeletons of vertebrates. We also suggest that more consideration should be given to investigating the patterning interaction between odontodes and dermal bones, both in the oropharynx and the skin, and that chondrichthyans (and thelodonts) that lack such bones are of limited value as models for understanding the general principles and evolution of odontode patterning. However, all these ideas should be tested by future examination of relevant fossil material, which is the only physical evidence documenting the origin of teeth [45].

## Supporting Information

Figure S1 **Further comparison of synchrotron scanning data and traditional thin section data. A.** A longitudinal virtual thin section from the synchrotron scanning data, red rectangle marking the region in **B**. **B.** Close-up of **A**, showing details of dentine tubules and enamel layer. **C.** Close-up of a real thin section of *Andreolepis* scale (PMU 24785), showing details of dentine tubules and enamel layer. **Abbreviations: bc,** basal canal penetrating the bony base; **dt**, dentine tubules; **e**, enamel; **hc**, horizontal vascular canal, **pc,** pulp cavity; **Shb,** Sharpey’s fibers.
(TIF)

Movie S1 **Original slices from the scan data of the** ***Andreolepis*** **scale (PMU 24786).**
(WMV)

Movie S2 **More information on the distribution and morphology of odontodes.**
(WMV)
