# Supplementary figures and images for: Scales and Tooth Whorls of Ancient Fishes Challenge Distinction between External and Oral ‘Teeth’
Source: PLoS One. 2013 Aug 12;8(8):e71890. doi: 10.1371/journal.pone.0071890 (PMC3741376; doi:10.1371/journal.pone.0071890)

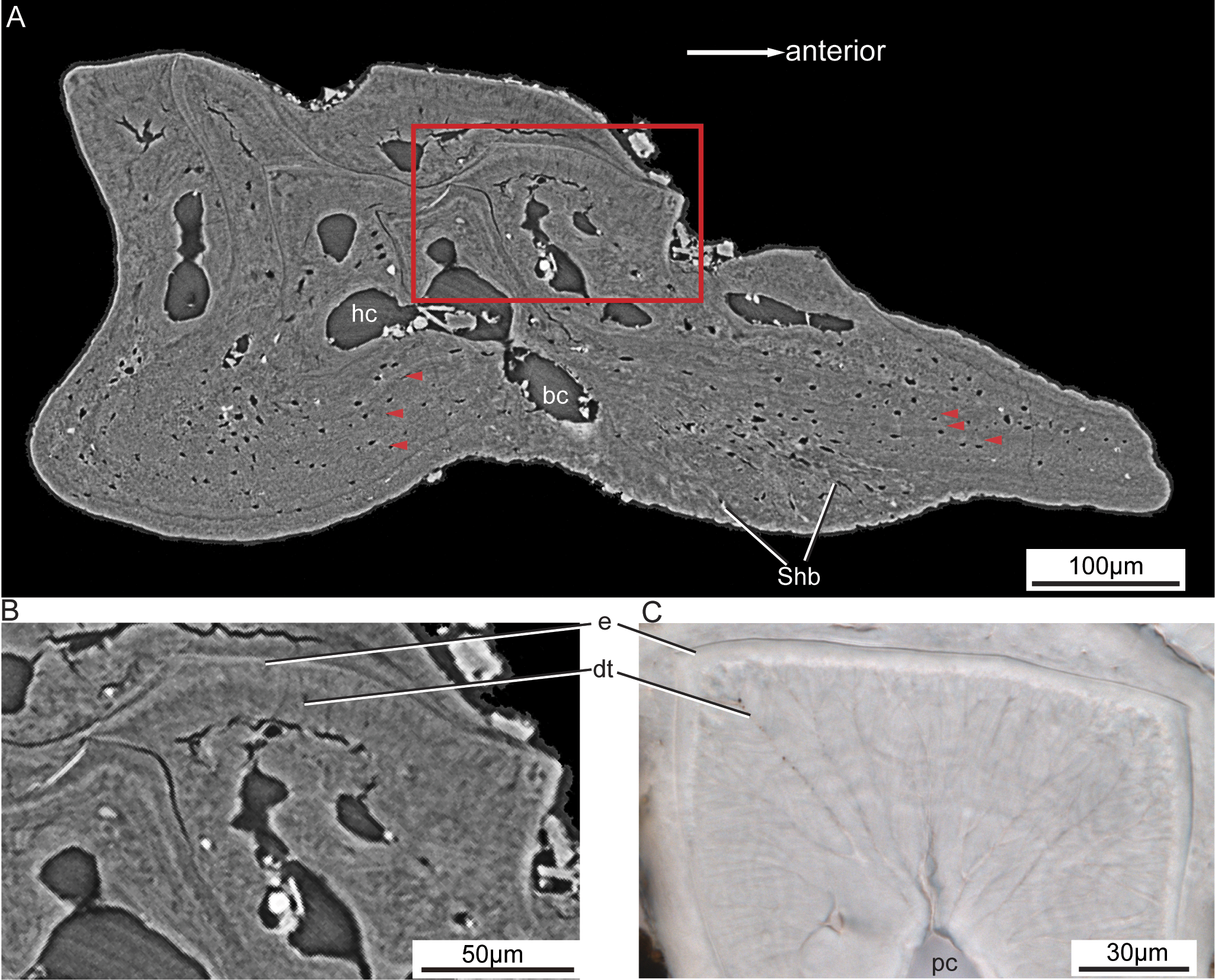

Supplement: Figure S1 — Further comparison of synchrotron scanning data and traditional thin section data. A. A longitudinal virtual thin section from the synchrotron scanning data, red rectangle marking the region in B. B. Close-up of A, showing details of dentine tubules and enamel layer. C. Close-up of a real thin section of Andreolepis scale (PMU 24785), showing details of dentine tubules and enamel layer. Abbreviations: bc, basal canal penetrating the bony base; dt, dentine tubules; e, enamel; hc, horizontal vascular canal, pc, pulp cavity; Shb, Sharpey’s fibers. (TIF) [file pone.0071890.s001.tif]
